# Melanin-Based Contrast Agents for Biomedical Optoacoustic Imaging and Theranostic Applications

**DOI:** 10.3390/ijms18081719

**Published:** 2017-08-07

**Authors:** Dario Livio Longo, Rachele Stefania, Silvio Aime, Alexander Oraevsky

**Affiliations:** 1 Consiglio Nazionale delle Ricerche (CNR), Istituto di Biostrutture e Bioimmagini, Torino 10126, Italy; 2 Dipartimento di Biotecnologie Molecolari e Scienze per la Salute, Università degli Studi di Torino, Torino 10126, Italy; rachele.stefania@unito.it (R.S.); silvio.aime@unito.it (S.A.); 3 TomoWave Laboratories, Houston, TX 77081, USA; AAO@TomoWave.com

**Keywords:** melanin, optoacoustic imaging, photoacoustic imaging, nanoparticle, contrast agent

## Abstract

Optoacoustic imaging emerged in early 1990s as a new biomedical imaging technology that generates images by illuminating tissues with short laser pulses and detecting resulting ultrasound waves. This technique takes advantage of the spectroscopic approach to molecular imaging, and delivers high-resolution images in the depth of tissue. Resolution of the optoacoustic imaging is scalable, so that biomedical systems from cellular organelles to large organs can be visualized and, more importantly, characterized based on their optical absorption coefficient, which is proportional to the concentration of absorbing chromophores. Optoacoustic imaging was shown to be useful in both preclinical research using small animal models and in clinical applications. Applications in the field of molecular imaging offer abundant opportunities for the development of highly specific and effective contrast agents for quantitative optoacoustic imaging. Recent efforts are being made in the direction of nontoxic biodegradable contrast agents (such as nanoparticles made of melanin) that are potentially applicable in clinical optoacoustic imaging. In order to increase the efficiency and specificity of contrast agents and probes, they need to be made smart and capable of controlled accumulation in the target cells. This review was written in recognition of the potential breakthroughs in medical optoacoustic imaging that can be enabled by efficient and nontoxic melanin-based optoacoustic contrast agents.

## 1. Introduction

Optoacoustic (OA) or Photoacoustic (PA) imaging is an imaging technique that provides high spatial resolution of optical contrast in the depth of optically scattering biological tissue [1,2,3]. Following nanosecond laser irradiation in the near-infrared spectral range, the localized increase in temperature results in a transient thermoelastic expansion that launches acoustic waves that are detected by ultrasound transducers. The detected optoacoustic signals are then converted into images using tomography algorithms [4,5]. The combination of optical and acoustic methods endows this hybrid imaging technique with higher spatial resolution and depth than optical imaging, but also with stronger tissue contrast than ultrasound alone. Thus, OA imaging can provide anatomical, functional, and molecular information in real time for investigating biological and pathological processes [6,7,8]. Endogenous molecules, such as hemoglobin of the blood and melanin, are the main tissue chromophores that can generate a stronger OA signal than other tissue components. Various applications have exploited endogenous chromophores for the visualization of the blood vasculature structure, for assessing tumor oxygenation, and for melanoma detection [9,10,11,12,13]. However, the endogenous contrast can be applied only in limited biological investigations with sub-optimal contrast capability. Therefore, the design of exogenous contrast agents may play a key role in improving contrast and imaging accuracy, and in providing specific molecular information [14,15]. To date, exogenous OA contrast agents are based on small-molecule dyes [16,17], carbon nanoparticles [18,19], or metallic nanostructures [20,21,22]. There are several reviews that summarize the development of PA exogenous agents with a general view [15,23,24].

Although exogenous nanoparticles can provide a strong contrast and higher specificity, toxicity and retention in the body are still the major obstacles for their clinical use. As a consequence, much attention has been devoted in the last years towards less toxic nanoparticles based on endogenous molecules [25,26]. Among them, melanin shows a broad optical absorption that makes it suitable for optoacoustic imaging. To date, several melanin-based imaging probes have been reported with specific properties suitable for optoacoustic imaging (Table 1). Optoacoustic contrast agents can be classified into two main categories: non-specific and specific probes. Non-specific probes simply extravasate and accumulate in tissue regions, according to their biodistribution and pharmacokinetic profiles. For tumor applications, these probes exploit the enhanced permeability and retention (EPR) effect that allows a non-specific accumulation within the tumor region [27]. Such a class of probes is extremely useful in detecting and delineating tumors, as well as in assessing tumor vasculature properties, such as permeability and vascular volume. Other contrast agents can be conjugated to molecular moieties (such as peptides or antibodies), providing active targeting capabilities, or be loaded with a drug, acting as drug carriers, or being exploited as therapeutic agents [28].

Within this review we will discuss the design, synthesis, physicochemical characterization, and optoacoustic imaging applications of different types of melanin-based imaging probes.

## 2. Melanin and Synthesis

**Scheme 1:**
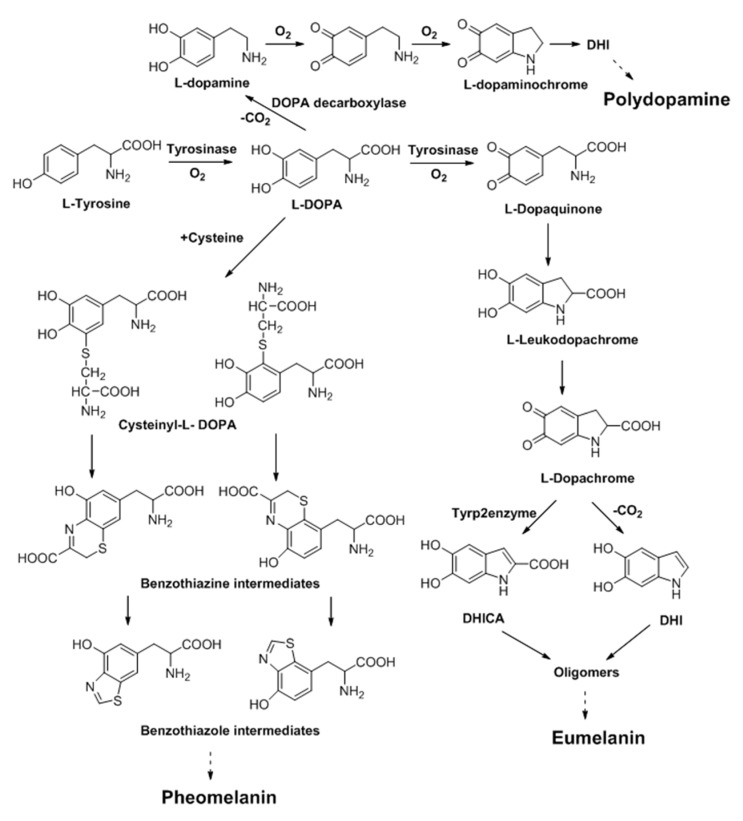
Synthetic pathways leading to eumelanin, pheomelanin, and polydopamine production.

Within the generic term “melanin”, a variety of biopolymers are found in nature as pigments in hair, skin, and eyes, obtained through a complex biosynthetic route known as melanogenesis [41]. The melanin structure is a macromolecular structure constituted of 5,6-dihydroxyindole (DHI) units, derived from the oxidative polymerization of dopamine, as well as of 5,6-dihydroxyindole-2-carboxylic acid (DHICA) units, derived from the oxidative polymerization of cysteinyl-dopamine. Subsequent oxidative polymerization of monomers DHI and DHICA via cross-link reactions give rise to dark/black insoluble melanin called eumelanin, and to reddish or light brownish melanin called pheomelanin (Scheme 1).

However, the organization of the constituent molecules of natural melanin, due to their poor water solubility, is still to be clarified, if formed by high-molecular weight polymeric chains or aggregates of oligomers [42,43,44]. Synthetic melanin-based nanoparticles are usually produced by auto-oxidation of dopamine (DOPA) in mildly alkaline aqueous solutions, or from auto-oxidation of dopamine, resulting in black insoluble synthetic forms of melanin called polydopamine [45,46].

## 3. Non-Specific Melanin-Based Probes for Tumor Imaging

Despite the fact that chemical and physical properties of natural melanin are preserved, synthetic melanins are different in shape and show low solubility in water [47]. Liopo and colleagues synthesized melanin nanoparticles with good dispersion stability in different biological media and increased their solubility throughout surface modification by PEGylation using thiol-terminated methoxy-polyethylene glycol [29]. The PEG-modified melanin nanoparticles showed no cytotoxicity up to 1.5 mg/mL (as measured by cell viability and cell lysis assays in human cell lines), which was two times lower than the toxicity of gold nanorods at the same optical density. The observed optoacoustic contrast at 800 nm was comparable to that of gold nanorods with equal optical density (1.0), and it was linearly correlated to the nanoparticle concentration. Following intravenous administration in mice (dose: 200 mg/kg b.w., concentration: 30 mg/mL), the nanoparticles showed a prolonged retention in the blood, with low uptake in the spleen and in the kidney regions due to their large size (ca. 50 nm) that prevents renal filtration and makes them invisible to the reticulo-endothelial system [48]. The results obtained in [29] demonstrated that improving melanin nanoparticle solubility is critically important in order to make optical absorbance competitive to that of red blood cells for biomedical imaging applications as optoacoustic contrast agents. Moreover, the exploitation of a 3D full view optoacoustic scanner, LOIS-3D, allowed authors to demonstrate an exceptionally high sensitivity (image contrast) in experiments performed in vitro and in vivo [4,49].

**Figure 1:**
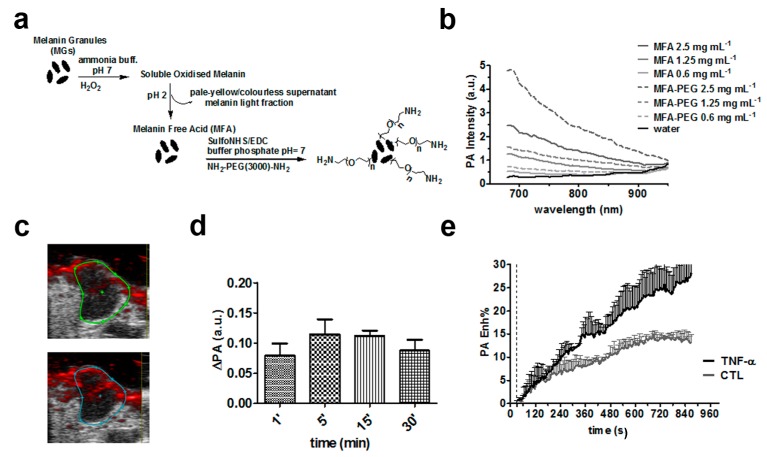
(**a**) Schematic illustration of the preparation of melanin-free acid derivatives (MFA) and their PEGylated form (MFA-PEG); (**b**) optoacoustic (OA) emission spectra of MFA and MFA-PEG at several concentrations in 1× PBS and pH = 7.4; (**c**) representative optoacoustic images in transverse section of tumor before (top) and 5 min after (bottom) intravenous injection of MFA-PEG (concentration: 2.5 mg/mL, volume: 0.1 mL) at 700 nm; (**d**) average OA signal changes in breast tumor-bearing mice (*n* = 4); and (**e**) averaged dynamic contrast-enhanced OA curves (OA Enh %) upon MFA-PEG tail vein injection for the control (*n* = 3) and TNF-α treated (*n* = 4) group, showing increased OA enhancement following antiangiogenic treatment. Reprinted with permission from reference [31], Copyright 2016, John Wiley and Sons.

Since melanin solubility is a key factor in determining optoacoustic efficiency, the capability to control its aggregation state is essential for future developments within the field of OA imaging and sensing [42,50]. Longo and colleagues developed highly soluble melanin derivatives starting from synthetic melanin granules by applying a slight bleaching procedure [31]. By treating melanin granules with a light oxidative process under neutral pH conditions, highly soluble melanin free acids (MFA) nanosystems were obtained still preserving the overall chemical nature of the pigment, without the need of PEGylation (Figure 1a).

The obtained MFA showed excellent stability, solubility and no in vitro toxicity by cell viability assays with J774 macrophage cell line up to a concentration of 2.5 mg/mL. At 700 nm a higher optoacoustic contrast was measured for the PEGylated MFA (MFA-PEG) in respect to MFA, both in vitro (Figure 1b) and in tumor bearing mice (Figure 1c,d) that may reflect the higher heat transfer efficiency between the nanosystem and water due to the PEG coating [51]. Interestingly, both MFA and MFA-PEG derivatives were small in size (7 and 11 nm, respectively), making them particularly suitable for assessing tumor vascularization properties. In fact, the dynamic acquisition of contrast enhanced images (DCE) over time after the intravenous injection of an exogenous contrast agent allows monitoring tumor vessel permeability [52,53]. DCE magnetic resonance imaging is an established approach that provides measurements of vascular functional changes and is considered a promising biomarker for assessing the drug treatment response [54,55,56,57]. For the first time, a dynamic contrast enhanced (DCE)-OA approach was exploited with melanin derivatives for assessing tumor vascular permeability in a breast tumor murine model. The time-intensity OA signal measured over time within the tumor region showed a constant uptake up to 15 min post MFA-PEG injection (dose: 10 mg/kg b.w., concentration: 2.5 mg/mL). Moreover, tumor vessel permeability changes were detected in a group of mice treated with TNF-α (100 μL of TNF-α at 10 μg/mL), a drug used in clinical trials that is known to increase vascular permeability (Figure 1e). This study clearly demonstrated that DCE-OA imaging is a suitable technique for assessing therapeutic treatment that targets the tumor vasculature.

Standard synthetic protocols yield polymeric particles with several types of supramolecular crosslinking that result in insoluble melanin polymers [43]. In a different approach for obtaining water-soluble melanin polymers without PEGylation, Repenko and colleagues reported the synthesis of a linear form of polydopamine [30]. Protecting the hydroxyl and the amine groups of dopamine during the synthesis to avoid crosslinking, polydopamine nanoparticles can be obtained into a linear form of dopamine with a low molecular weight. In contrast to the broad-band absorption spectra of natural melanin caused by the large number of electronic transitions coming from different subunits, these melanin nanoparticles showed two absorption bands due to their defined and linear structure [43]. An excellent optoacoustic contrast was observed at 700 nm, with a detection limit of this linear melanin ca. two orders of magnitude higher than that of the gold nanoparticles.

## 4. Responsive Melanin-Based Probes

**Figure 2:**
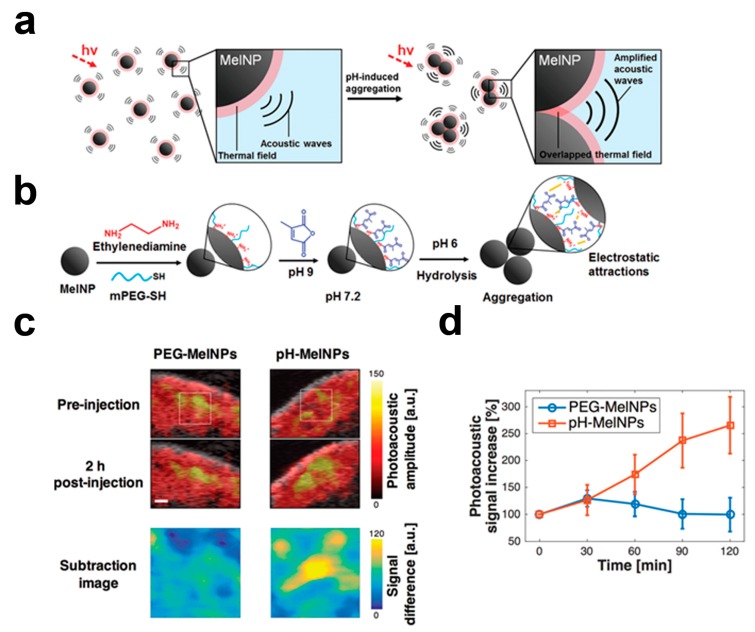
(**a**) Schematic illustration of the proposed aggregation-induced amplification of the OA signal from melanin nanoparticles (MelNP) upon laser irradiation (hv, dashed arrow); (**b**) the surface modification of the bare melanin nanoparticles with citraconic amide results in the aggregation, under mildly acidic conditions (pH-MelNP); (**c**) combined ultrasound (US, in gray) and optoacoustic (OA, in red and yellow) images of B16 melanoma in living mice before and after the intravenous injections (concentration: 1 mg/mL, volume: 0.2 mL) of the PEG-MelNP (left column images) and the pH-responsive melanin-nanoparticles (right column images). The difference image was obtained by subtracting OA signals in the tumor site (a white box) of the pre-injection image from those in the post-injection image; (**d**) a change in the difference of OA signal intensity between the pre- and the post-injection images acquired at 30, 60, 90, and 120 min. Reprinted with permission from reference [32], Copyright 2016, Royal Society of Chemistry.

In contrast to conventional contrast agents, responsive probes can change their OA signal, responding to the biomolecular targets or the event of interest [58]. The main advantage of this is related to improved imaging sensitivity and specificity, since it can only be activated by a specific interaction with or the recognition of the (bio)molecular partner. The counterweight of this approach is the more complex design and synthesis, limiting so far their broad development. Despite this limitation, several nanoprobes have been developed for assessing pH [59,60], reactive oxygen species [61,62], metal ions [63,64], and enzymatic activity [65,66,67] within the optoacoustic imaging field. Several approaches for reporting PA signal changes in response to the target of interest have been exploited, including enzyme cleavage, self-assembly and aggregation, and variations in molecular structure and conformation. Ju and colleagues reported the pH-induced aggregation of a melanin nanoparticle for monitoring tumor acidic microenvironment [32]. The surface of melanin nanoparticles was modified using citraconic anhydride, yielding a hydrolysis-susceptible citraconic amide in mildly acidic conditions with an average size of 130 nm (Figure 2a,b). Under pH 7, the hydrolysis of citraconic amide groups results in nanoparticles with both negative and positive surface charges that aggregate due to the electrostatic interactions. This physical aggregation of melanin nanoparticles induced a strong increase in the OA signal (ca. 9 times at 700 nm) after exposure to the mild acidic condition (pH = 6) in comparison to the neutral condition (pH = 7.2). The author examined the efficacy of this nanoparticle for specific in vivo tumor imaging, due to the acidic microenvironment that is usually found in solid tumors [68,69]. Tumor-bearing mice (B16f10 melanoma cell line) were injected with probes capable of pH responsible aggregation or with control nanoprobes not decorated with a citraconic amide (concentration: 1 mg/mL, volume: 200 µL). The PA signal from the tumor region increased up to 2.4 times in the mouse injected with the pH-sensitive nanoprobe at 2 h post-injection, whereas no signal increase was observed for the control nanoprobe, demonstrating the aggregation-induced amplification of the OA signal in the tumor site (Figure 2c,d). Despite the accumulation strategy, which suggested that exploiting aggregation processes may work well in tumors, further studies are needed to assess any toxicity effect following accumulation in other organs exposed to acidic conditions, such as lungs and kidneys, where aggregated particles may remain for long times.

## 5. Targeted Melanin-Based Probes

Accumulation of OA imaging probes delivered intravenously is usually achieved by exploiting the EPR effect, which is less efficient (with a lower accumulation) when compared to active targeting. Targeted PA imaging probes provide specific information on cellular and molecular targets exploiting the overexpression of the receptor or the antigens. The surface of the probes is composed of targeting moieties, such as antibodies, peptides, and small-molecule ligands that can bind with high affinity and specificity for overexpressed receptors, thus enabling the cellular and molecular imaging of cancer. Several targeted PA agents have been developed, based on functionalization with antibodies [16,70,71], peptides [72,73], or small molecules [28,74,75]. Owing to the presence of quinine groups in the melanin structure, simple modification of the melanin particle is feasible by reaction with thiols or amines via Michael addition or Schiff base reactions, enabling the introduction of targeting moieties [45]. Tumor growth and metastasis are heavily dependent on the angiogenic process that induces expression of molecules in endothelial cells, including integrins, which modulate cell adhesion and migration [76]. α_v_β_3_ integrins are highly upregulated during angiogenesis, providing a molecular target for delivery of imaging probes directly to the vasculature of growing tumors [52,77]. The arginine-glycine-aspartic acid (RGD) sequence in the proteins of the extracellular matrix binds α_v_β_3_ integrins; thus, imaging probes conjugated to cyclic RGD (cRGD) peptides enable the evaluation of α_v_β_3_ integrin expression in tumor and endothelium cells.

**Figure 3:**
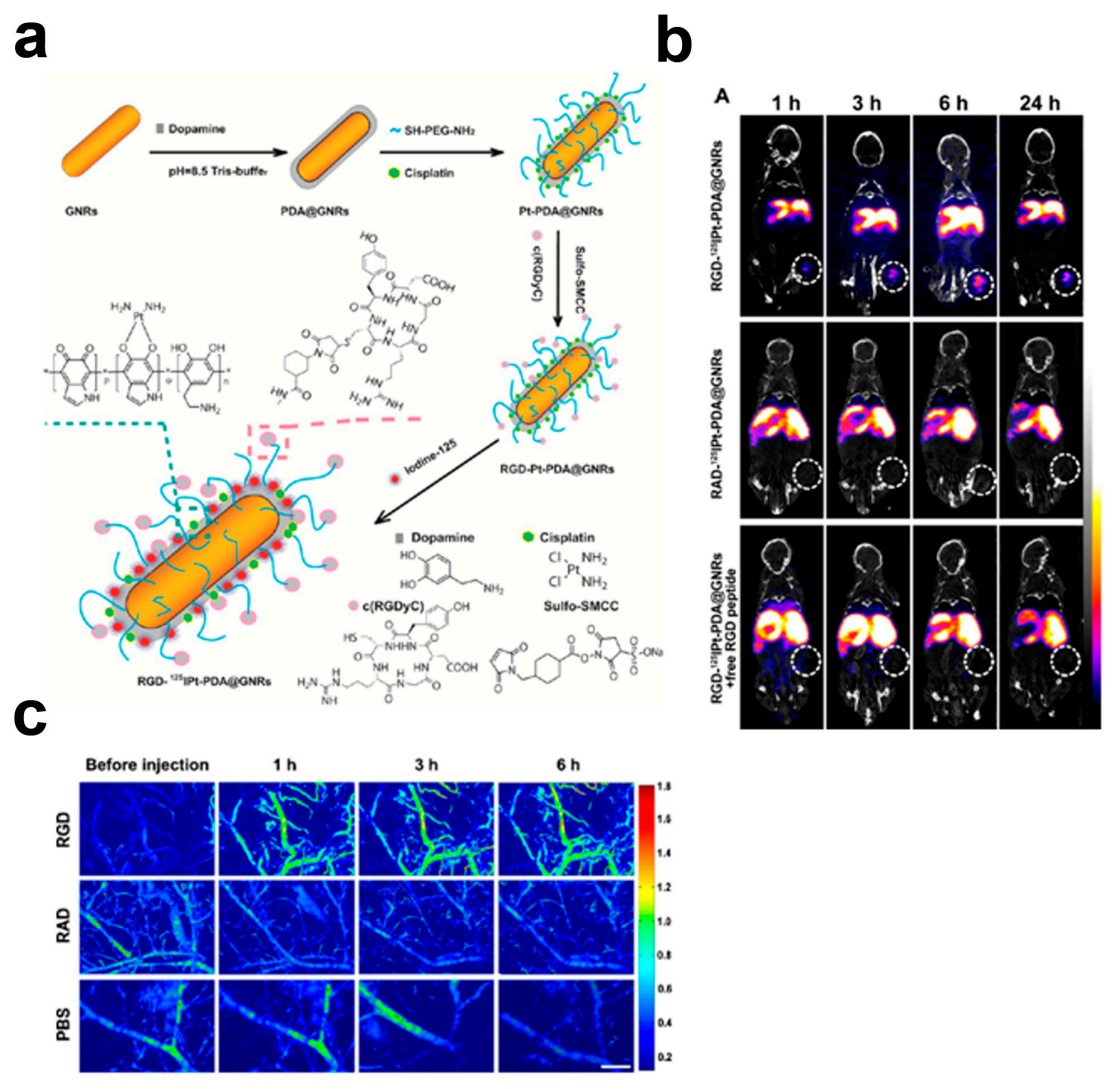
(**a**) Schematic illustration of the polydopamine (PDA)-coated gold nanorods (GNR) labeled with ^125^I, loaded with cisplatin (Pt), and conjugated to the arginine-glycine-aspartic acid (RGD) peptide targeting α_v_β_3_ integrins; (**b**) single-photon emission computed tomography/computed tomography imaging of tumor-bearing mice after intravenous injection with RGD-^125^IPt-PDA@GNRs, RAD-^125^IPt-PDA@GNRs (non-targeting peptide), or RGD-^125^IPt-PDA@GNRs probes plus free RGD peptide (100 μL, 0.15 mM), at a radiation dose of 300 μCi, showing the targeting specificity of the RGD peptide to the α_v_β_3_ integrins; (**c**) optoacoustic imaging of tumors after intravenous injection with RGD-^125^I-Pt-PDA@GNRs (RGD) at a dose of 40 mg Au/kg b. w., and RAD-^125^IPt-PDA@GNRs (RAD) as a control. Scale bar = 10 μm. Reprinted with permission from reference [35], Copyright 2016, American Chemical Society.

Gambhir’s group reported a targeted melanin-based probe for α_v_β_3_ integrins overexpressed in a glioma murine model (U87MG glioma cell line) [33]. A cyclic RGDfC (arginine-glycine-aspartic acid-d-phenyl-cysteine) peptide was conjugated to ultrasmall PEGylated melanin nanoparticles (ca. 8 peptides per nanoparticle) that, upon intravenous injection (concentration: 200 µM, volume: 200 µL) in U87MG tumor mice, showed an increased optoacoustic signal at 4 h post injection. This enhanced PA contrast can be attributed to the cumulative contributions of the EPR effect and the tumor targeting capability to α_v_β_3_ integrins. To further confirm the active targeting property of the developed melanin-based probe, a control peptide, with a non-targeting property for α_v_β_3_ integrins, was conjugated to a PET-^64^Cu-melanin nanoparticle. The observed stronger PET signal obtained with the cRGDfC-conjugated peptide in the tumor at 4 h post-injection, in comparison to the control peptide, clearly indicated the specific targeting properties of the cRGDfC-PEG-melanin nanoparticle, allowing a higher tumor accumulation capability. Similarly, Zhang and colleagues studied a targeted melanin-decorated nanoprobe for imaging α_v_β_3_ integrins expression in lung murine tumors [35]. Gold nanorods were polydopamine coated, and PEGylated and the cRGD peptide were covalently coupled through thiol-maleimide linkages with ca. 58 cRGD peptides immobilized per the gold nanorods molecule (Figure 3a). The specificity of the cRGD-probe was evaluated in α_v_β_3_ integrins positive human H1299 cells (non-small lung cancer cell line) and compared to that of the gold nanorods conjugated to a control peptide. The cellular uptake of the cRGD probe was higher than that of the control peptide and the uptake was significantly reduced by the free RGD peptide, thus indicating that the probes were specifically targeting α_v_β_3_ integrins-positive cells. Studies performed in tumor-bearing mice with the targeted nanoprobe labeled with ^125^I for SPECT imaging were acquired following injection of the RGD peptide, or conjugated to a control peptide (non-targeting α_v_β_3_ integrins), or with an excess of the free peptide, demonstrating the high specificity towards integrins also in vivo (Figure 3b). Tumor-bearing mice injected intravenously with the cRGD-polydopamine-gold nanorods (dose: 40 mg Au/kg b.w.,) showed an augmented PA signal in the blood vessels of mice, revealing the detailed vascular structure of the tumor (Figure 3c). These observations confirmed the specific targeting of tumor angiogenic vessels, since gold nanorods conjugated with the control peptide showed no increase in OA contrast. A different peptide, arginine-glycine-aspartic-cysteine acid (RGDC), was exploited for targeting α_v_β_3_ integrins overexpressed in tumors by Li and colleagues [34]. The RGDC peptide was grafted onto the surface of the polydopamine nanoparticles throughout covalent linking to the termini of the PEG chains that were then bound onto the surface of polydopamine via the Schiff/Michael reaction. The specific interaction between the RGDC moiety and α_v_β_3_ integrins was assessed in HeLa cells, although no control peptide was exploited for further confirmation of the binding specificity. In vivo studies in HeLa tumor-bearing mice showed a consistently higher PA contrast in the tumor at 8 h in comparison to the non-targeted polydopamine nanoparticles (dose: 20 mg/kg b.w.,).

## 6. Multi-Modality Melanin-Based Probes

**Figure 4:**
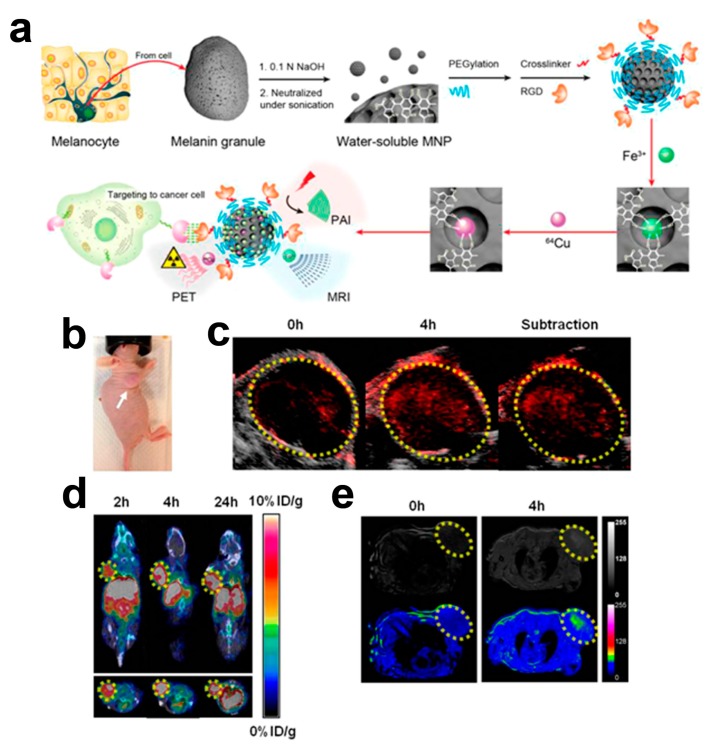
(**a**) Schematic illustration of the synthesis of the multimodality melanin nanoparticles (MNP). After PEG surface-modification, RGD peptides were further attached to the melanin-nanoparticles for tumor targeting. Then Fe^3+^ and/or ^64^Cu^2+^ were chelated to the obtained MNPs for OA/MRI/PET multimodal imaging; in vivo multimodality imaging in U87MG tumor-bearing mouse: (**b**) photographic image (white arrow refers to the tumor position); (**c**) overlaying of ultrasonic (gray) and optoacoustic (red) imaging; (**d**) overlaying of decay-corrected coronal (top) and transaxial (bottom) small animal CT (gray) and PET (color) images; (**e**) MRI images (top row shows black and white images, and bottom row shows the pseudocolored images) after tail vein injection ^64^Cu-Fe-RGD-PEG-MNP (tumor region is enveloped by yellow dotted line). Reprinted with permission from the reference [33], Copyright 2014, American Chemical Society.

In comparison to single modality alone, multimodal imaging (by combining different imaging modalities) can provide complimentary information and exploit intrinsic advantages of each modality. In particular, magnetic resonance imaging (MRI) provides high spatial resolution and soft tissues contrast; fluorescence/optical imaging (OI) has high sensitivity but limited tissue penetration; ultrasound imaging (US) provides real time monitoring and is easy to operate; computed tomography (CT) provides high anatomical imaging but limited functional information; and positron emission tomography (PET) has high sensitivity and provides biochemical information [78,79,80,81]. Several nanomaterials have already been investigated for multimodality imaging, including gold-iron oxide nanoparticles and single-walled carbon nanotubes for MRI and OA [28,82,83], gold nanoparticles for OA and CT [84,85], quantum dots for OA and OI [86], and gold microbubbles or perfluorocarbon nanodroplets for OA and US [87,88]. The synthesis of multimodality nano-platforms is usually complicated by the need to integrate several contrast properties (by using different reporting moieties or by chemical modifications) into a single entity throughout time-consuming processes and difficult chemical routes. An important property of melanins is related to their capability to strongly chelate metal ions (Fe, Cu) [89,90]. Fan and colleagues exploited this natural binding ability towards metal ions to develop a multimodality nanoplatform for PA, MRI (Fe^3+^), and PET (^64^Cu^2+^) with simplified building process [33]. Melanin nanoparticles were obtained with the assistance of sonication (to decrease aggregation, hence improving solubility) with ultra-small size (ca. 5 nm) and stability. Long PEG chains (PEG5000) were introduced to further improve water solubility of Fe^3+^ and Cu^2+^ chelated melanin nanoparticles (Figure 4a). A high loading capacity was obtained, with 100 and 90 ions per one melanin nanoparticle, for Cu^2+^ and Fe^3+^, respectively. Fe^3+^-loaded melanin nanoparticles (MRI T_1_ contrast agent) showed modest contrast efficiency (R_1_ of 1.2 mM^−^1·s^−1^), but were sufficient to provide a 30% contrast enhancement following intravenous administration (concentration: 200 µM, volume: 200 µL) in U87MG tumor-bearing mice. In the same murine tumor model, ^64^Cu^2+^-loaded melanin nanoparticles (PET imaging) showed a gradual tumor uptake increase up to 24 h with a clear tumor contrast after 2 h post-injection. By mixing melanin nanoparticles with both Fe^3+^ and ^64^Cu^2+^ to form the multifunctional probe, they demonstrated tumor contrast enhancement properties in all the three imaging modalities (PA, PET, and MRI, Figure 4b–e), albeit using large doses (concentration: 200 µM, volume: 250 µL). These results showed that melanin nanoparticles can be exploited as an active platform for in vivo multimodality imaging, combining its native optoacoustic properties with radioactive and magnetic ones that provide molecular and anatomical information at high spatial resolution.

Hong and colleagues investigated similar nanoprobes for multimodal imaging (PA/MRI/PET) by loading in melanin nanoparticles Gd^3+^ cations for MR applications [38]. PEGylated melanin nanoparticles were successfully loaded with ca. 50 Gd^3+^ atoms per molecule without any chelator ligand, for a final size of 15 nm. As free Gd^3+^ is toxic, no release from nanoparticles was observed in buffered solutions at different pH values up to 48 h, even in the presence of excess metal chelator DOTA, thus suggesting high stability. The relaxivity of these Gd-loaded nanoparticles was ca. two times higher (R_1_ of 6.8 mM^−1^·s^−1^) than that of the clinical Gd-based contrast agent, Gd-DOTA. Following tail vein injection in healthy mice (concentration: 8 mM, volume: 100 µL), a significant signal intensity increase was measured in the liver, suggesting that the elimination route is through the hepatobiliary system. The PET imaging probe was easily obtained by simple mixing ^64^Cu^2+^ with the melanin nanoparticles, with a radiolabeling yield of 85%. The in vivo PET images confirmed the liver accumulation of the ^64^Cu-melanin nanoparticles, paralleled by OA images. Despite melanin nanoparticles simplifying the preparation of multimodal probes without using chelator ligands, some concerns about the possible long-term toxicity following the free Gd^3+^ ions release need to be investigated.

Ju and colleagues reported Fe^3+^-loaded melanin nanoparticles with improved MRI contrast efficiency [39]. Synthetic melanin nanoparticles were prepared with an average size of ca. 100 nm, conjugated with thiol-terminated methoxy-poly(ethylene glycol) and treated with an Fe^3+^ solution, yielding a very high longitudinal relaxivity (R_1_ of 17 mM^−^1·s^−1^), ca. three times higher than that of the clinically-approved, Gd-based contrast agents [91,92]. Such a marked increase in the MRI contrast efficiency is likely explained by the restricted rotational mobility of the complex, due to the nanoparticle size and the contribution of the so-called outer sphere relaxation [93,94]. Positive signal enhancements were observed in T_1_-weighted MR images in the spleen and liver, upon intravenous administration of PEGylated Fe^3+^-loaded melanin nanoparticles (dose: 0.020 mg/kg b.w.,) in healthy mice, because of the accumulation in the reticuloendothelial system.

Fluorescence imaging is a technique extensively used in the biological lab, owing to high sensitivity, the fact it is relatively inexpensive, and because it can simultaneously monitor several molecular events, but is limited by low spatial resolution and reduced sensitivity with an increased imaging depth [40]. Therefore, combing optical imaging with optoacoustic imaging may expand the applicability of non-invasive imaging approaches. Xiao and colleagues have designed and tested melanin carbonaceous dots as a new structure for dual modality imaging (optical plus optoacoustic) [95]. Melanin carbonaceous dots prepared using a hydrothermal method (size ca. 40 nm) showed a specific absorbance peak at 630 nm and a fluorescence emission peak at 660 nm that render them suitable for both optical and optoacoustic imaging [96]. When intravenously injected in 4T1 tumor bearing mice (dose: 66.3 mg/kg b.w.), the fluorescence signal increased from 2 h up to 24 h, indicating a long circulation time in the blood stream and tumor accumulation due to the enhance permeability and retention effect. Optoacoustic imaging performed at the same time points showed similar results, with significant accumulation up to 24 h post injection.

## 7. Theranostic Melanin-Based Probes

The combination of diagnostic and therapeutic agents on a single platform, dubbed theranostic, is emerging as a novel therapeutic approach for treating cancer. Nanoparticles have an intrinsic advantage in combining imaging and therapeutic functionalities into the same platform, enabling an increased diagnostic accuracy and improved therapy efficacy [97]. However, major concerns are related to the long-term safety of several nanomaterials due to poor biocompatibility and biodegradability [98]. Consequently, biocompatible theranostic nanosystems are urgently needed to foster their clinical applications and translation. Polydopamine nanoparticles have been intensively investigated due to their excellent biocompatibility and as a versatile platform for different imaging and therapy strategies.

PDA nanoparticles can be exploited as chemotherapy agents owing to their ability to bind drugs by π-π stacking interactions due to the presence of aromatic rings on their surface, or by hydrogen bonding [99]. Zhang and coworkers prepared ultra-small melanin nanoparticles as a drug-loading system for sorafenib [37], an FDA-approved multikinase inhibitor for hepatocellular carcinoma [100]. This nanosystem allows the delivery of lower dosages of the drug, hence reducing its side effects. The high drug-loading capacity can be explained by the high surface to volume ratio provided by the nanoparticle. Moreover, a gradual and linear release of the drug induced a high cytotoxicity against HepG2 cancer cells. The PA properties were slightly affected after sorafenib loading on melanin nanoparticles, and following intravenous injection into mice bearing HepG2 xenograft (dose: 40 mg/kg b.w.,), the PA signal was still detectable at 24 h post injection, indicating that the drug-loaded melanin nanoparticles were retained inside the tumor. A higher antitumor effect was observed when compared to the drug alone, due to the prolonged circulation of the nanoparticles and to the sustained delivery of sorafenib within the tumor, thus markedly enhancing the safety profile of sorafenib for tumor treatment.

Chemotherapy, as well as surgery and radiotherapy for treating cancers, are limited by the potential risks of damaging normal cells, impairing the immune system, or increasing the incidence of secondary cancers. Photothermal therapy (PTT) is an emerging technique for treating cancer, owing to minimal invasiveness and high selectivity [101]. The advantages of this technique rely on the localized effect at the tumor site following agents accumulation and on the non-ionizing near-infrared (NIR) laser exposure, reducing considerably the risks of conventional clinical cancer therapies. Penetration of NIR light through tissues is determined by several factors, including: wavelength, beam diameter, and the effective optical attenuation coefficient (dependent on tissue composition). In general, longer wavelengths (up to 1070 nm) will penetrate deeper due to gradually decreasing optical scattering. On the other hand, local peaks of the optical absorption in lipids (930 nm) and water (980 nm) can result in slightly a lower penetration depth compared with that at about 800 nm. The number of optical photons effectively acting upon tissues at a given depth is a linear function of the incident optical fluence (in the pulsed regime) or the optical power (in the CW regime), i.e., parameters limited by the laser safety standards established by the American Laser Institute [102]. The effective density of optical photons decreases approximately three times with every 10 mm penetration of NIR light in tissue [3]. Thus, only a small fraction of incident optical energy can actually reach deeply situated tumors, which makes the PTT of cancer impossible without the use of contrast agents. As a consequence, various materials have been explored as NIR-light absorbers in order to increase the thermal energy released in the targeted cancer tissues.

**Figure 5:**
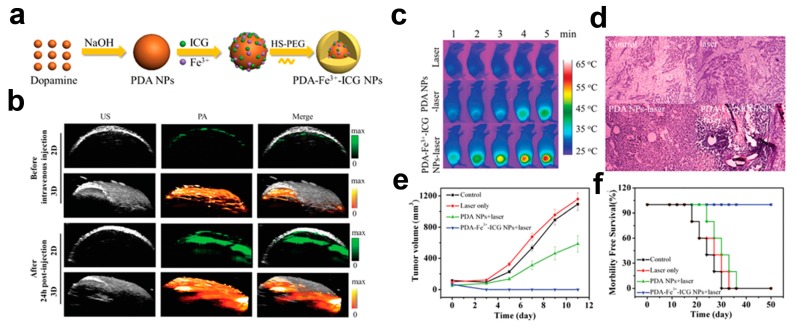
(**a**) Schematic illustration of the preparation process of polydopamine nanoparticles (PDA-NPs) loaded with indocyanine green (ICG) and labeled with Fe^3+^; (**b**) in vivo OA images of the PDA–Fe^3+^–ICG NPs in a 4T1-bearing tumor mice model; in Vivo photothermal therapy (PTT) in 4T1-bearing tumor mice showing (**c**) IR thermal images of 4T1 tumor-bearing mice exposed to 808 nm laser for 5 min (1.0 W·cm^−2^); (**d**) H&E stained images of tumor sections collected from the different treated groups of mice 6 h post-treatment; (**e**) tumor growth curves for the different groups of 4T1 tumor-bearing mice; (**f**) survival rates of the mice bearing 4T1 tumors after the different treatments. The data indicate the mean and standard errors (*n* = 5). Reprinted with permission from reference [36], Copyright 2016, Royal Society of Chemistry.

Since melanin-like biopolymers show wide optical absorption properties, they can be exploited as agents for the photothermal therapy of cancer [103]. In addition, these naturally occurring substances can effectively avoid serious adverse effects caused by long-term retention and the lack of biodegradation of other metal-based nanoparticles. However, polydopamine nanoparticles show a low absorption coefficient in the NIR region, thus requiring high-power density lasers (808 nm, 2 W·cm^−2^) that may induce thermal damage to normal tissues. To overcome this limit, Hu and colleagues developed a novel PTT agent based on dopamine-melanin colloidal nanospheres incorporating the FDA-approved indocyanine green (ICG) dye [36] for dual-modal imaging and PTT treatment (Figure 5a).

The incorporation of the ICG dye resulted in a six-fold increase of the NRI optical absorption, hence improving both optoacoustic contrast and photothermal conversion efficiency. The i.v. injection (dose: 40 mg/kg b.w.,) in a breast tumor murine model (4T1 breast cancer cell line) resulted in marked PA contrast up to 24 h post-injection, reflecting the passive accumulation in the tumor, with a blood circulation half-life of ca. 3 h (Figure 5b). Upon irradiation with a low NIR laser pulse (808 nm, 1 W·cm^−2^) for 5 min, the efficient translation of the laser energy to heat induced a significant killing of cancer cells (Figure 5c,d). The tumor growth in the laser-treated groups was not affected by the laser irradiation alone, but a partial suppression was observed when polydopamine nanoparticles were administered, whereas a complete tumor growth inhibition was obtained upon administration of the ICG-loaded polydopamine nanoparticles (Figure 5e,f).

A further improvement in the strategy for cancer treatment is obtained by combining both photothermal therapy and chemotherapy [104]. In fact, chemotherapy suffers from the serious side effects and drug resistance, while photothermal therapy suffers from incomplete tumor ablation due to the heterogeneous distribution of hyperthermia. Therefore, the combination of both therapeutic approaches should achieve an increased therapeutic efficiency [105]. Since the penetrating NIR radiation can be exploited simultaneously for optoacoustic imaging and for heat generation, this offers the possibility to exploit single melanin-based nanoparticles. Li and colleagues showed that biodegradable polydopamine nanoparticles are excellent platforms for OA imaging, PTT therapy, and for the loading anticancer drug (doxorubicin) [34]. Polydopamine nanoparticles were synthesized by oxidation and the self-polymerization of dopamine, pegylated on the surface and conjugated to a RGDC peptide, for in vivo targeting of α_v_β_3_-positive tumors through the binding to RGD-α_v_β_3_ integrins [106]. This system exhibited broad absorbance in the NIR region with high photothermal conversion efficiency (39%). Doxorubicin was successfully loaded through hydrophobic interactions, with a drug release triggered by a low pH environment, as found in the interstitial space of tumors [69]. A specific interaction was observed between the RGD moiety and the human hepatic cancer HeLa cells, overexpressing α_v_β_3_ integrins, and resulting in a higher cellular uptake than that of the non-targeting PDA. In addition, the highest rate of cell death was observed following NIR irradiation, in comparison to single treatment (chemotherapy or photothermal therapy) conditions. In mice bearing the subcutaneous HeLa tumor, a consistent high optoacoustic signal was observed at 8 h post-intravenous injection (dose: 20 mg/kg b.w.,) due to the binding to tumor cells or to the vasculature. To further demonstrate this nanoprobe for tumor theranostic, in mice bearing HeLa tumors a rapid increase in temperature (52 °C) was observed upon laser irradiation, causing irreversible damage to cancer cells. Moreover, the tumor was completely eradicated without regrowth in the combination (chemo + photothermal) therapy.

Since melanin can also be exploited as an optimal coating material, due to highly stable drug loading and chelator-free metals labeling, Zhang and colleagues developed a novel targeted and theranostic imaging probe based on gold nanorods decorated with melanin for efficient chemotherapy (cisplatin) and SPECT (^125^I) imaging [35]. Gold nanorods (GNR), synthesized with an average size of 50 × 15 nm^2^, were coated with a uniform melanin layer (1–2 nm thick) by self-polymerization of dopamine under alkaline conditions. Further modifications allowed us to: (i) conjugate PEG5000 on the surface of these nanoparticles; (ii) load cisplatin drug; (iii) conjugate the arginine-glycine-aspartic acid (RGD) peptide for targeting αvβ3 integrins; and (iv) label with radioisotope ^125^I for SPECT imaging (Figure 3c). The integrity of the melanin coating was demonstrated at different biological conditions and upon irradiation, with no morphological changes detected even at high temperature (90 °C) upon photothermal conversion of GNR. Under acidic conditions a sustained release of cisplatin was observed, up to 50% at 24 h and pH = 6. Treatment of H1299 tumor-bearing mice with these theranostic nanoparticles (cisplatin dose = 2.5 mg/kg b.w.; nanoparticle dose: 40 mg Au/kg b.w.; irradiation 0.5 W/cm^2^, 808 nm) resulted in tumor shrinking and elimination already at three days post-treatment, with any tumor relapse up to 21 days.

All these studies validate the use of melanin-based nanoparticles for theranostic imaging, showing an outstanding synergistic effect on tumor treatment when combining photothermal therapy with chemotherapy.

## 8. Summary and Outlook

Most of the applications for which melanin-based probes have been developed so far focus on oncology diseases. The OA probes summarized herein have been designed to provide information on tumor vasculature permeability, receptor expression, and tumor microenvironment. Moreover, they were exploited for multimodality imaging, combining anatomical and functional information, and for drug delivery and therapy. Melanin was demonstrated to be a versatile material to provide an optoacoustic signal, either as a platform for imaging agents, or exploited to decorate other kinds of nanoparticles for OA contrast generation. In addition, the useful binding capabilities of melanin allow us to easily design OA probes with a drug/metal ion loading capability and to further functionalize the nanoprobe by covalent linking of other moieties (e.g., peptides for active targeting or PEG chains for improving solubility).

Despite the progress that melanin-based probes for OA imaging have demonstrated in the last decade, challenges are still to be overcome to broaden their use in basic research and to facilitate translation from preclinical to clinical settings. Due to the inherent low solubility of natural melanin, several synthetic procedures have been pursued to increase water solubility and dispersibility of melanin-based OA probes. However, considering that the ability to provide the OA signal is dependent on the polymeric/aggregation state and the size of melanin structure, a trade-off between solubility and OA contrast capability is required. A deeper understanding of the structure-property-function relationships is therefore crucial to design melanin-based probes with improved OA contrast properties [50]. Another important aspect is the size of the probe that has a strong impact on biodistribution properties, elimination routes, and half-lives. Considering that the size of the reported melanin-based OA probes cover a broad range (5–150 nm), much effort is still needed to tailor probe size with specific applications. For clinical applications, smaller size should be preferable, since rapid clearance avoids long-term accumulation of the probe, hence reducing safety concerns. Melanin is a biodegradable component, hence safety for melanin-based probes should not be an issue, but further functionalization that adds metals (e.g., gadolinium) or that decorates other nanomaterials (e.g., gold nanorods) for providing multimodality properties needs to be properly characterized for metal release, since no chelators are used for metal binding. There is a significant need for standardized procedures for characterizing these nanomaterials, as well as for protocols for their use in animal models, in order to improve multi-site comparison. Furthermore, to date, no standard protocols have been established to assess long term stability, accumulation, biodistribution, and safety that are mandatory to promote translation between basic research and clinical applications. Thus, future work should aim to synthesize probes with higher optoacoustic contrast efficiency, higher biocompatibility, and optimized circulation times.

Although melanin-based probe development is just beginning, these reported studies have demonstrated promising opportunities for improving imaging, diagnosis, and therapy.

## Figures and Tables

**Table 1:** Summary of melanin-based contrast agents for optoacoustic imaging.

Contrast Agent	Description	Size (nm)	Target	Principal Applications	Ref.
PEG-MNP	Pegylated polydopamine nanoparticles	50		3D OA vascular imaging	[29]
PEG-MNP	Linear polydopamine nanoparticles	-		OA vascular imaging	[30]
PEG-MNP	Melanin nanoparticle	7–10		Assessing tumor vasculature and response to antiangiogenic therapy	[31]
PEG-MNP-CA	Pegylated polydopamine nanoparticles decorated with citraconic amide	130	Tumor acidic microenvironment	OA cancer imaging	[32]
^64^Cu-Fe-cRGDfC-PEG-MNP	Pegylated polydopamine nanoparticles conjugated to cyclic RGD peptide and loaded with ^64^Cu^2+^ and Fe^3+^	5–10	α_v_β_3_ integrins	OA cancer imaging of integrins and multimodality imaging (OA/PET/MRI)	[33]
DOX-RGDC-PEG-MNP	Pegylated polydopamine nanoparticles conjugated to cyclic RGD peptide and loaded with doxorubicin	120	α_v_β_3_ integrins	OA cancer imaging of integrins and chemo-photothermal therapy of tumors	[34]
^125^I-Pt-cRGDyC-PEG-MNP-GNR	Gold nanorods decorated with pegylated polydopamine nanoparticles conjugated to cyclic RGD peptide and loaded with cisplatin and ^125^I	54	α_v_β_3_ integrins	OA cancer imaging of integrins, chemo-photothermal therapy of tumors and multimodality imaging (OA, SPECT)	[35]
ICG-Fe-PEG-MNP	Pegylated polydopamine nanoparticles loaded with indocyanine green and Fe^3+^	146		Photothermal therapy of tumors and multimodality imaging (OA, MRI)	[36]
SRF-PEG-MNP	Pegylated polydopamine nanoparticles loaded with sorafenib	60		Imaging-guided chemotherapy	[37]
Gd-^64^Cu-PEG-MNP	Pegylated polydopamine nanoparticles loaded with Gd^3+^ and ^64^Cu^2+^	15		Multimodality imaging (OA/MRI/PET)	[38]
Fe-MNP	Polydopamine nanoparticles loaded with Fe^3+^	98		Multimodality imaging (OA/MRI)	[39]
MNP-CD	Melanin-decorated carbonaceous dots	40		Multimodality imaging (OA/OI)	[40]

## Abbreviations

PA	Photoacoustic (Optoacoustic) Imaging
NIR	Near Infra Red
NP	Nanoparticles
PDA	Polydopamine
MNP	Melanin nanoparticles
PEG	Polyethylene glycol
PET	Positron Emission Tomography
SPECT	Single Photon Emission Computed Tomography
MRI	Magnetic Resonance Imaging
CT	Computed Tomography
OI	Optical Imaging
US	Ultrasound
b.w.	body weight
